# Phenylephrine per se or Combined with Pregabalin Ameliorates Mononeuropathic Pain in Rats

**DOI:** 10.3390/pharmaceutics18030334

**Published:** 2026-03-08

**Authors:** Sarah Kadhim Abbood, Nariman Essmat, Imre Boldizsár, Judit Mária Kirchlechner-Farkas, Csenger Kovácsházi, Yashar Chalabiani, Kornél Király, Ildikó Miklya, Zoltán Giricz, Laszlo G. Harsing, E. Sylvester Vizi, Mahmoud Al-Khrasani

**Affiliations:** 1 Department of Pharmacology and Pharmacotherapy, Faculty of Medicine, Semmelweis University, Nagyvárad tér 4, H-1089 Budapest, Hungary; abbood.sarah@phd.semmelweis.hu (S.K.A.); nariman@zu.edu.eg (N.E.); boldizsar.imre2@semmelweis.hu (I.B.J.); kirchlechner.farkas.judit.mari@semmelweis.hu (J.M.K.-F.); kovacshazi.csenger@semmelweis.hu (C.K.); chalabiani.yashar@semmelweis.hu (Y.C.); kiraly.kornel@semmelweis.hu (K.K.); miklya.ildiko@semmelweis.hu (I.M.); giricz.zoltan@semmelweis.hu (Z.G.); harsing.laszlo@semmelweis.hu (L.G.H.J.); 2 Center for Pharmacology and Drug Research & Development, Semmelweis University, Üllői út 26, H-1085 Budapest, Hungary; 3 Department of Clinical Pharmacy and Therapeutics, Faculty of Pharmacy, University of Kufa, Najaf 54001, Iraq; 4 Department of Pharmacology and Toxicology, Faculty of Pharmacy, Zagazig University, Zagazig 44519, Egypt; 5 Molecular Pharmacology Research Group, Hun-Ren Institute of Experimental Medicine, Szigony utca 43., 1083 Budapest, Hungary

**Keywords:** phenylephrine, noradrenaline release, pregabalin/phenylephrine combination, neuropathic pain

## Abstract

**Background/Objectives:** Neuropathic pain (NP) affects approximately 6.9–10% of the population and is inadequately managed by the current therapies, as reflected by a high number needed to treat (NNT). These data highlight the socio-economic burden of NP on healthcare. Thus, the repurposing of existing medications and new drug combinations to enhance therapeutic efficacy are required. **Methods/Results:** Here, we show that intrathecal phenylephrine (PE) in a dose of 3, 10, or 30 nmol/rat acutely alleviates tactile allodynia in rats with mononeuropathic pain evoked by partial sciatic nerve ligation. Prazosin and idazoxan, which are considered as selective α_1_- and α_2_-adrenoreceptor antagonists, respectively, reversed the antiallodynic effects of PE. In ex vivo experiments, PE induced a significant cytosolic [^3^H]-noradrenaline release from mouse spinal tissue. In addition, in the mouse vas deferens, PE produced smooth muscle contraction in prazosin and idazoxan sensitive manner. As a novelty, in another set of experiments, oral PE (5 mg/kg) and pregabalin (PGB, 25 mg/kg) combination, but not the individual drug treatments, acutely alleviated allodynia in rats with mononeuropathy. In addition, the antiallodynic action of the combination was further enhanced upon chronic treatment. Under isoflurane anesthesia, this combination was devoid of cardiovascular side effects attributed to systolic and diastolic blood pressure, mean arterial pressure, or heart rate. PGB induced motor dysfunction was not altered upon the combination with PE. **Conclusions:** These data suggest that PE in combination with PGB shows promise in preclinical settings; however, the necessity for further studies is paramount to detail the pharmacokinetic interactions involved.

## 1. Introduction

Neuropathic pain (NP) is defined by the International Association for the Study of Pain as pain caused by a lesion or disease of the somatosensory nervous system [1]. Epidemiologically, NP affects approximately 6.9–10% of the population [2,3,4]. According to multiple international societies, the first-line medications for NP include systemic tricyclic antidepressants (TCAs), serotonin–norepinephrine reuptake inhibitors (SNRIs), and gabapentinoids (gabapentin and pregabalin) [2,5,6,7,8,9,10]. Topical lidocaine (5%) and capsaicin (8%) patches are recommended as second-line therapies. However, these topical patches can be considered as first-line therapy for patients who cannot tolerate the systemic first-line therapies or have focal NP [2,5,7,10]. Analgesic drugs possessing both opioid and non-opioid mechanisms of action, such as tramadol, are recommended as second-line therapeutic options, while classical strong opioids are reserved as third-line pharmacological therapies for the management of NP [2,5,7,8]. The failure to treat NP successfully by the current therapies is reflected by the low response rate of the patients; for instance, the response rate for first-line medications like TCAs is 50%, while this rate is 30–50% in the case of other drugs, such as low-dose opioid analgesics or the 8% capsaicin patch [2,6,11]. The unsatisfactory nature of monotherapy-based treatments is also reflected by a high and variable number needed to treat (NNT), indicating that adequate NP control has not yet been fully achieved [5]. For instance, the NNT for TCAs (amitriptyline, imipramine, and clomipramine), norepinephrine transporter (NET) inhibitors (nortriptyline and desipramine), gabapentinoids (gabapentin and pregabalin), and SNRIs were found to be 2.1, 2.5, 3.9 and 5, respectively [7,12,13]. In placebo-controlled trials, the combined NNT for 50% pain relief value for TCAs, SNRIs, pregabalin (PGB), and gabapentin was 3.6, 6.4, 7.7, and 6.3, respectively [5]. In addition, the current pharmacological therapies are associated with a significant adverse effect profile, which can limit their clinical utility, specifically when dose escalation is followed to obtain better efficacy. These data point to the fact that the limitation of the current therapy to treat NP is multifaceted and arises from modest and variable efficacy of most pharmacological approaches with substantial tolerability burdens. For instance, the current TCAs display notable anticholinergic and cardiotoxic risks, which constrain use and dosing, especially in high-risk populations. In the case of SNRIs, the cardiotoxic side effect is lower compared to TCAs. Gabapentinoids, further first-line medications, also have many side effects. The most common adverse effects of PGB are dizziness and somnolence, followed by dry mouth, edema, and blurred vision, with somnolence being the cause of about 4% of treatment discontinuations. Dizziness and somnolence are also common side effects of gabapentin, along with confusion and peripheral edema, which are dose-related and reversible. Opioid analgesics, besides their conventional side effects such as constipation (which remains a constant problem due to no development of tolerance), nausea, vomiting, respiratory depression, cognitive impairment, and finally addiction liability, can also contribute to increased mortality due to unintentional overdose. The number needed to harm of these currently used therapies (especially those used in first-line) is low, reflecting that the treatment of NP is not only limited by the severity but also the prevalence of their adverse effects, alongside their low efficacy and slow onset of action [5,14,15,16,17].

Furthermore, the high rate of non-responders remains a considerable burden on the socio-economic well-being of the general population suffering from NP [4,18,19]. Concerning economic burden, it is important to note the dual nature of costs: direct, including medical and non-medical ones, and indirect, associated with lost time from work [11,20,21,22].

Combining drugs with different pharmacodynamic profiles has aimed to improve NP relief and decrease side effects in recent decades; however, none have provided satisfactory treatment thus far [5,23,24].

Consequently, a significant unmet need remains for the development of novel treatment strategies, whether mono- or combination-based, for managing NP conditions. As such, the current work centres on the potential of repurposing existing medications that are used to treat other ailments within clinical settings to address the management of NP.

Phenylephrine (PE) is a synthetic sympathomimetic agent with potent vasoconstrictive properties; it is predominantly described as a selective α_1_-adrenoceptor (α_1_-AR) agonist [25,26]. It is used as a vasopressor in clinical practice to treat acute hypotension in patients experiencing shock or hypotension brought on by anesthesia [27,28]. Contrary to the current canonical standpoints, our group has recently uncovered the existence of a substantial indirect effect of PE, specifically highlighting the role of [Ca^2+^]-independent non-vesicular noradrenaline (NA) release in mediating the effects of PE on the contraction of the smooth muscle of the mouse vas deferens (MVD) [29]. In addition, the effect of PE was found to be highly sensitive to nisoxetine, a selective inhibitor of the NET, suggesting that PE acts as a NET substrate, reverses transporter function, consistent with previous observations [29,30,31,32,33,34].

Considering the pharmacological aspect of some current first-line drugs, specifically those that regulate NA reuptake by increasing the level of NA in the spinal cord, such as TCAs and SNRIs, an innovative strategy based on this mechanism is still worth investigating. NA displays high affinity to α-ARs, and endogenous NA plays a crucial role in the inhibition of NP [35,36]. Importantly, drugs that activate α_2_-ARs like clonidine or dexmedetomidine (analgosedatives) inhibit NP evoked by spinal nerve ligation in animals [35,37,38,39].

Considering the spinal cord distribution of α-ARs, namely α_1_ and all subtypes of α_2_, including α_2_A and α_2_B, except α_2_C, it is evident that they are activated by NA. In fact, the α_2_B-AR subtype has been reported to be involved in neurotransmission in the spinal cord, thereby presenting significant implications for the treatment of NP [40].

As mentioned above, the current monotherapies for treating NP have a high NNT and a slow onset of action [5,41]. To enhance the analgesic efficacy of a current single drug treatments like PGB, dose escalation is required, but this comes at the high risk of side effects [42,43]. Thus, the search for novel mono- and combination-based therapies to manage NP has been pursued to develop a treatment with high efficacy, rapid onset of action, and a low incidence of side effects [17,44,45,46,47,48,49,50].

PGB, an anticonvulsant that modulates the α_2_δ subunit of voltage-gated calcium channels and is approved by the FDA for the treatment of diabetic neuropathy, has been tried in combination with other drugs in the hope of obtaining an effective treatment approach for NP, while being devoid of the unwanted side effects [48,51,52,53,54].

The hypothesis underlying our current research is based on observations that PE can induce cytosolic NA release, as previously described [29,30,31,32,33,34]. This neurochemical observation raises an intriguing possibility: if PE indeed causes this release in the spinal cord, then injecting PE into the spinal cords of rodents, such as rats or mice, might lead to pain relief through α-AR mechanisms. The α_2_-AR-mediated antinociception has been well documented in the existing literature [39,55].

Therefore, the aim of the current study is to investigate the effect of PE in rats with NP. To this end, the following studies should be conducted:

The antiallodynic impact of PE in rats with NP will be assessed. A model of peripheral mononeuropathic pain induced by partial sciatic nerve ligation (pSNL) will be utilized and then rats with NP will receive intrathecal doses of PE. In the same pain model, the investigation will focus on the receptors that mediate the effects of intrathecal PE. Ex vivo experiments to examine the impact of PE on NA release from spinal tissues will also be conducted. If the expected results are obtained, further studies will evaluate the systemic antiallodynic effect of PE alone and in combination with PGB, a first-line medication currently used in clinical practice. Finally, side effects related to motor and cardiovascular functions will be measured for PE, PGB, and their combination.

## 2. Materials and Methods

### 2.1. Animals

Male Wistar rats (170–250 g), male CD1 (24–30 g) and NMRI mice (35–45 g) were used. Male Wistar rats and NMRI mice were housed at the local animal facility of the Department of Pharmacology and Pharmacotherapy at Semmelweis University in Budapest, Hungary. Male CD1 mice were kept in the Hun-Ren Institute of Experimental Medicine, Hungary. Male Wistar rats and NMRI mice were purchased from Toxi-Coop Zrt. in Budapest, Hungary. Male CD1 mice were obtained from the local house, with approval from the local Animal Care Committee (PE/EA/285-5/2020). The study was conducted in accordance with the ARRIVE guidelines. Animals were placed in standard cages with five animals per cage and kept in a room at a temperature of 20 ± 2 °C with a 12 h light/12 h dark cycle. Water and food were ad libitum.

### 2.2. Drugs

The test compounds phenylephrine (PE), prazosin, idazoxan, and clonidine were obtained from Sigma-Aldrich (St. Louis, MO, USA). Pregabalin was kindly provided as a gift by Meditop Pharmaceuticals Ltd. (Budapest, Hungary). Levo-[7-^3^H]-noradrenaline (specific activity = 20 Ci/mmol) was purchased from American Radio-labelled Chemicals (St. Louis, MO, USA). Prazosin and clonidine were dissolved in distilled water, whereas PE, idazoxan and PGB were dissolved in 0.9% saline. In experiments intended to test the effects of intrathecal PE, prazosin and idazoxan on rats with NP, the test drugs were administered in a volume of 5 µL/rat by using a Hamilton syringe injected between L4 and L5. In experiments intended to investigate the effect of oral PE and PGB, drugs were administered by orogastric gavage (5 mL/kg). For anesthetic purposes, pentobarbital 60 mg/kg dissolved in 0.9% saline and administered intraperitoneally in a volume of 2.5 mL/kg. Pentobarbital was delivered from Semmelweis University Pharmacy, Budapest, Hungary. The constituents of Krebs’ solution were obtained from REANAL labor, Budapest, Hungary.

### 2.3. The Rat Partial Sciatic Nerve Ligation Pain Model

Rats underwent pSNL, as previously described by Seltzer and utilized by our group to induce mononeuropathic pain in rats [48,56]. Briefly, the rats (120–170 g) were placed on a cushion at 30 °C after being anesthetized with 60 mg/kg of pentobarbital administered intraperitoneally. The operator carefully exposed the sciatic nerve of the right hind paw at the level of the thigh without causing any muscle damage under aseptic conditions. After that, A 6-0 polypropylene suture was used to tightly ligate the sciatic nerve so that the dorsal 1/3 to 1/2 of the nerve thickness was captured in the ligature. Two stitches were used to seal the wound. Rats used as controls underwent sham surgery, leaving the nerve intact.

The experimental schedules 1 and 2 involved an NP model with pre-experiment acclimatization and subsequent pain assessments. After two days of animal handling, the rat basal paw withdrawal thresholds (PWTs) were measured, and sciatic nerve operations were performed after that. The rat PWTs were assessed by DPA (dynamic plantar Aesthesiometer 37450; Ugo Basil, Italy) 5 min following the cage acclimation period. According to the manufacturer’s instructions, the DPA apparatus alternately raises a metal filament with a diameter of 0.5 mm to the right and left hind paws, with forces ranging from 1 to 100 g (cutoff). The average of the three PWT measurements taken on each paw was employed for subsequent analysis. On the 14th day following surgery, DPA was used to measure tactile allodynia, which is one of the hallmarks of NP signs. Allodynia was defined in each rat as a 20 per cent drop in the average PWT value of the operated (right) paw compared to the unoperated (left) paw, as previously described [48,56,57]. After measuring the basal PWTs, PE, prazosin, idazoxan or vehicle was administered intrathecally in amounts of 5 µL per rat, and PWTs were measured 15, 30, and 60 min following treatments again (experimental schedule 1). Sham-operated animals were used as controls. In all experiments shown in experimental schedules 1, 2 and 3, animals were randomized before the treatment by an online random generator, and Group allocation was unknown during the experimental procedures. Finally, the group size was decided according to our previous studies [41,48].


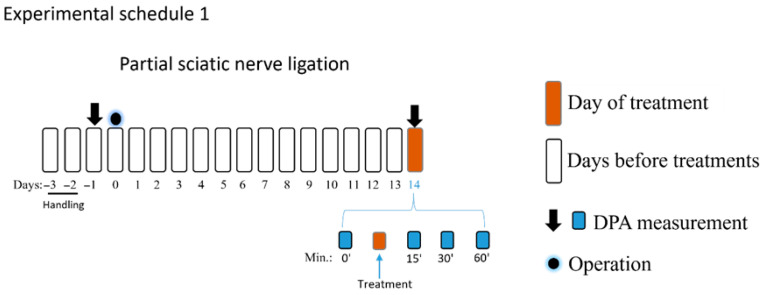


In the second part of the study (experimental schedule 2), on day 14, the PWT-baseline were first measured, followed by intrathecal treatment with PE (30 nmol) alone or in combination with prazosin (5 and 10 nmol) or idazoxan (10 nmol), prazosin (10 nmol) alone, idazoxan alone (10 nmol), or vehicle. Thereafter, PWTs were measured at 30 min. The chosen time to measure the impact of prazosin and idazoxan on the effect of PE was based on the first part of the study, as PE achieved a peak effect at this time point.


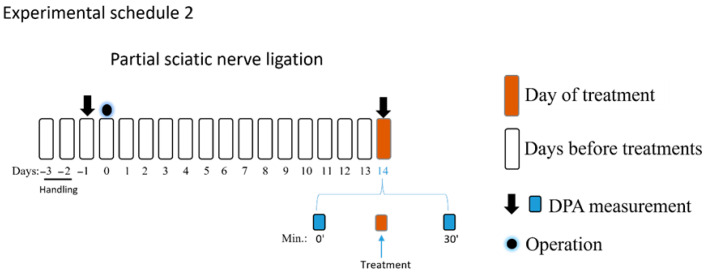


The third part of the study aimed to determine the effect of oral PE (5 mg/kg) per se, PGB (25 mg/kg) per se, PE/PGB (5 and 25 mg/kg) or PE/PGB (5 and 12.5 mg/kg). The effect of PE, PGB, the combination, or vehicle was measured 60 and 120 min after oral administration. Thereafter, the treatments with test drugs or the test combination were continued for an additional 7 days, administered twice a day, and on day 21, the effects of the test drugs and combination were measured 60 and 120 min following oral administration (experimental schedule 3).


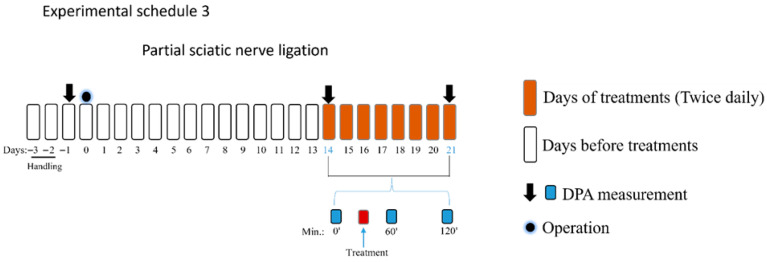


The dosages for the combination therapy were selected based on established sub-therapeutic thresholds and clinical equivalency. PGB was administered at doses of 12.5 mg/kg and 25 mg/kg, which were previously shown to be ineffective as monotherapy in this model [48]. PE dose of 5 mg/kg was determined using body surface area normalization, as described previously [58]. This dose is equivalent to approximately 56 mg in humans, which aligns with clinically relevant parameters. Consequently, the study evaluated the oral administration of 5 mg/kg PE in combination with either 12.5 mg/kg or 25 mg/kg PGB.

### 2.4. In Vitro Measurement of [^3^H]-Noradrenaline Release from Spinal Cord

#### 2.4.1. Preparation of Spinal Cord Slices

[^3^H]-noradrenaline ([^3^H]NA) release were performed in male CD1 mouse spinal cord slices. Noradrenergic fibres originating from the A5, A6 and A7 regions are the main sources of spinal NA [59]. Briefly, mice were euthanized following the American Veterinary Medical Association’s Guidelines for the Euthanasia of Animals: 2020 Edition (available online: https://www.avma.org/resources-tools/avma-policies/avma-guidelines-euthanasia-animals (AVMA, 2020, access date: 6 March 2026). The procedure involved initial anesthesia with >1.5% isoflurane in a sufficiently large chamber to ensure rapid loss of consciousness and prevent hypoxia, followed by decapitation. The thoracolumbar parts of the spinal cord were immediately removed and immersed in ice-cold, carbogen-saturated (95% O_2_, 5% CO_2_) Krebs solution in mM: NaCl, 118.0; NaHCO_3_, 25.0; KCl, 4.7; KH_2_PO_4_, 1.2; glucose,11.0; CaCl_2_, 2.5; and MgSO_4_, 1.2 [60].

#### 2.4.2. Calculation of [^3^H]-Noradrenaline Release

Mouse spinal cord slices (400 μm thick) were prepared using a tissue chopper and incubated for 45 min in 1 mL of Krebs solution containing [^3^H]NA at a final concentration of 3 μCi. To investigate the effects of PE, the drug was added to the perfusion medium and maintained throughout the experiment. Following three washes with 10 mL of ice-cold, oxygenated Krebs solution, the slices were transferred to a two-channel microvolume (100 μL) perfusion system maintained at 37 °C [61]. Each chamber contained four slices, which were superfused with Krebs solution at a rate of 0.5 mL·min^−1^ for 60 min before measurements. This relatively low flow rate was chosen to limit dilution and preserve released transmitters, consistent with prior studies [61,62,63]. [^3^H]NA release was quantified over 19 consecutive 3 min fractions. Electrical field stimulation (Grass S88 stimulator: 20 V, 2 Hz, 2 ms for 2 min) was applied during the third (S1) and thirteenth (S2) fractions. After each experiment, 500 μL of superfusate from each fraction was mixed with 2 mL of Ultima Gold scintillation cocktail (Packard). Tissues were then removed, and residual radioactivity was extracted in 5 mL of 10% trichloroacetic acid over 30 min. A Packard 1900 Tricarb and 5110 TR liquid scintillation counter was used to measure the radioactivity in both the superfusate and extracts. Results were expressed as disintegrations per minute per gram wet tissue weight (Bq g^−1^) as described previously [64]. The total release of radioactivity over the resting release (R1 and R2, respectively) was used to compute the electrical field stimulation (S1 and S2)-induced release. The average radioactivity release in the first and second fractions was denoted by R1, and the average radioactivity release in the fourteenth and fifteenth fractions by R2.

### 2.5. Mouse Vas Deferens Assay

Vasa deferentia were prepared using the previous protocol, with minor adjustments [65]. Briefly, vasa deferentia were removed from NMRI mice (35–45 g), unsheathed and suspended between an upper (ring) and a lower (straight) electrodes in 5 mL organ baths containing Krebs solution [29]. The organ baths were aerated with a gas mixture consisting of 95% oxygen and 5% carbon dioxide. The upper end of the MVD was attached to a transducer that processes the contractions for a computer through an amplifier. The basal tension of the isolated organ was brought to 0.1 g. The contractions were induced by an electrical field stimulation of the following parameters: trains of 10 Hz with 10 rectangular impulses at a pulse width of 1 ms and an intensity of 9 V/cm, considered supramaximal [29]. The stimulation was repeated at a frequency of 0.1 Hz using the Stimulator 88 device from Grass Medical Instruments, located in Quincy, MA, USA [29]. With the use of a thermoelectric device (Frigomix 2000, B. Braun Biotech International, Melsungen, Germany), the temperature of the Krebs solution was kept constant at 36 °C.

Two series of experiments were carried out. In the first series of experiments, the contractions of individual vasa deferentia were electrically induced and left for 50–60 min equilibration to ensure the ability of each organ to respond to stimuli. Thereafter, the electrical stimulation was stopped and the MVD contractions were induced by 10 µM PE. In this part of the experiments, the effect of PE was investigated alone or in the presence of 3 μM prazosin or 1 and 3 μM idazoxan, or vehicle. PE was allowed to sit for 2 min before being washed. Then, after 5–6 washes, prazosin or idazoxan was added to the organ bath. It was then allowed to equilibrate for 10 min before PE was injected once again.

In the second series of experiments, the organs, similar to the first series of experiments, were adjusted for 50–60 min of equilibration. Then, the effect of clonidine (1 μM) on the electrically induced MVD contractions was evaluated in the presence of 1 μM idazoxan.

### 2.6. The Rotarod Test

The rotarod test was used to assess motor coordination in male naïve Wistar rats. For this purpose, the Rat Rotarod (Model 7750; Ugo Basile, Gemonio, Italy) was employed. Animals were trained to remain on the apparatus’s rotating rod for 180 s (cutoff time) one day before the experiment, with the instrument’s speed set to 16 rpm as previously described [48]. On the next day, after the oral treatment with PE (5 mg/kg), PGB (25 mg/kg), the PE and PGB combination (5 mg/kg + 25 mg/kg), or vehicle, the rat motor coordination was measured at 60 and 120 min in accordance with results obtained in rats with mononeuropathy. The latency time, also known as the fall-off time, was measured to evaluate motor function.

### 2.7. In Vivo Assessment of Blood Pressure and Heart Rate

Naïve male Wistar rats were anesthetized with 5% induction and 1.5% isoflurane maintenance. The right carotid artery was cannulated, and blood pressure and heart rate were measured continuously from 60 to 120 min after oral drug administration, as described earlier [66] using AD Instruments devices (AD Instrument, Bella Vista, Australia). Physiological animal core temperature was maintained using a heating pad (Harvard Apparatus, Holliston, MA, USA). Animals were randomized for the treatment. To analyze systolic, diastolic, mean arterial pressure and heart rate, 30 s of measurement was averaged every 10 min. The analyzer was blinded to the treatment groups. The heart rate was derived from the ECG signal.

### 2.8. Statistical Analysis

The data were analyzed using GraphPad Prism 8.0 Software, a statistical analysis application based in San Diego, CA, USA. The data were presented as the mean ± standard error of means (SEM). The data underwent one-way or two-way ANOVA analysis, followed by Dunnett’s test for conducting multiple comparisons. The Kruskal–Wallis test with uncorrected Dunn’s post hoc test was also used. For the release experiments, a two-tailed *t*-test was applied. Statistically significant differences were considered to exist if the *p*-value was less than 0.05. A ROUT method analysis was performed to identify any outliers using a Q value of 1%. The normality of the data distribution was assessed using the Shapiro–Wilk test, which has high power across a range of sample sizes. For data meeting the assumption of normality and homogeneity of variance, parametric tests (e.g., Student’s *t*-test or ANOVA) were employed. In cases where data significantly deviated from normality (*p* < 0.05), non-parametric equivalents (Kruskal–Wallis test) were employed to maintain analytical robustness.

## 3. Results

### 3.1. Intrathecal PE Alleviates Tactile Allodynia in Rats with Mononeuropathic Pain

**Figure 1:**
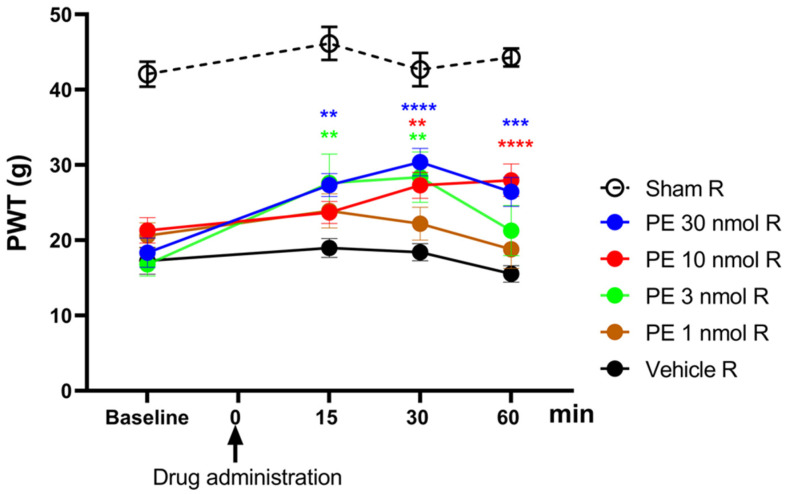
The acute antiallodynic effects of intrathecal PE in rats with pSNL. The impact of the test doses of 1, 3, 10, and 30 nmol PE or vehicle was examined 15, 30, and 60 min following administration. PWTs of operated right (R) hind paws or sham-operated R on day 14 after operation are presented in grams. Data are given as mean ± SEM at the indicated time points, *n* = 5–13 per group. The PWTs were compared before and after treatment (two-way ANOVA F (15, 209) = 1.979; Dunnett’s post hoc test, ** *p* < 0.01, *** *p* < 0.001, **** *p* < 0.0001 statistically significant compared to the vehicle-treated right (R) operated hind paw. Number of rats used per group: sham (*n* = 6), vehicle (*n* = 13), 30 nmol PE (*n* = 9), PE 10 nmol (*n* = 9), PE 3 nmol (*n* = 5), and PE 1 nmol (*n* = 6).

The acute antiallodynic effect of intrathecally administered PE at the following doses: 1, 3, 10, and 30 nmol/rat was tested in rats exhibiting significant tactile allodynia, on day 14 after pSNL (Figure 1). PE in doses of 3, 10, and 30 nmol/rat produced a significant antiallodynic effect at 30 min following administration. Notably, PE at a dose of 30 nmol produced a significant antiallodynic impact at every time point and peaked at 30 min after administration. Therefore, this dose was used for further investigations. Given the potential for changes in the central nervous system in chronic pain that may affect PWTs on both sides, the contralateral side was not utilized as a control to rigorously eliminate this bias. Therefore, the PWTs of the right paws of animals that underwent pSNL and displayed tactile allodynia were used to measure the impact of the vehicle.

### 3.2. Intrathecal Co-Administered Prazosin Inhibits the Antiallodynic Effects of PE in Rats with Mononeuropathic Pain

**Figure 2:**
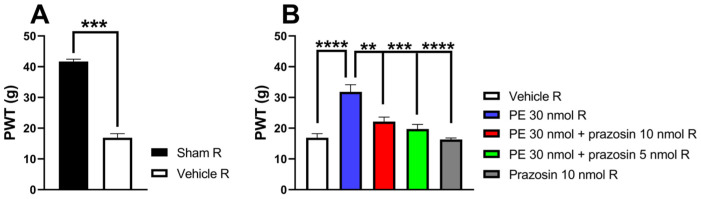
(**A**) The two-tailed *t*-test with the Mann–Whitney test was used to compare the pain threshold between the sham-operated R and the vehicle R group (Mann–Whitney test, U = 0, *p* = 0.0003). (**B**) The acute antiallodynic effects of PE (30 nmol/rat), administered intrathecally in the presence of 5 or 10 nmol/rat prazosin on day 14 after pSNL. The impact of the test drugs was investigated 30 min after administration. Data are given as mean ± SEM, *n* = 4–12 per group. Tukey’s post hoc test with one-way ANOVA was used to analyze the PWTs (** *p* < 0.01, *** *p* < 0.001, **** *p* < 0.0001), F = 13.5. Number of rats used per group: sham (*n* = 5), vehicle (*n* = 12), 30 nmol PE (*n* = 6), 30 nmol PE + 10 nmol prazosin (*n* = 5), 30 nmol PE + 5 nmol prazosin (*n* = 7) and 10 nmol prazosin (*n* = 4).

To investigate whether the AR subtypes mediate the antiallodynic effect of PE on day 14 after pSNL on the right paw exhibiting allodynia, PE was administered at a dose of 30 nmol per rat. Intrathecal PE (30 nmol/rat) significantly elevated PWTs, demonstrating its antiallodynic effect 30 min post-administration, as evidenced by the previously mentioned experiments (Figure 1). Co-administration of prazosin (selective α_1_-AR antagonist) at 5 and 10 nmol/rat inhibited the antiallodynic effect of PE (Figure 2). The administration of either prazosin alone or vehicle failed to produce an impact on PWTs.

### 3.3. Intrathecal Co-Administered Idazoxan Inhibits the Antiallodynic Effects of PE in Rats with Mononeuropathic Pain

**Figure 3:**
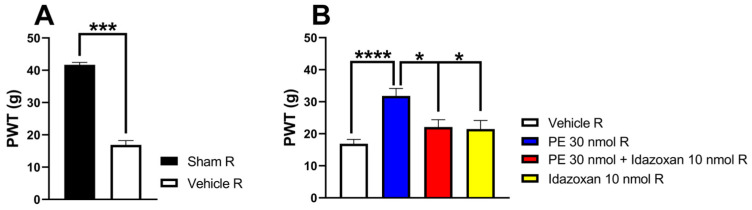
(**A**) The two-tailed *t*-test with the Mann–Whitney test was used to compare the pain threshold between the sham-operated R and the vehicle R group (Mann–Whitney test, U = 0, *p* = 0.0003). (**B**) The acute antiallodynic effects of intrathecal PE (30 nmol/rat) alone or in the presence of 10 nmol/rat idazoxan in rats on day 14 after pSNL. The PWTs were measured 30 min after administration of the test compounds. Data are expressed as mean ± SEM, *n* = 5–12 per group. The PWTs were compared using one-way ANOVA with Tukey’s post hoc test (* *p* < 0.05, *** *p* < 0.001,**** *p* < 0.0001), F = 10.33. Number of rats used per group: sham (*n* = 5), vehicle (*n* = 12), 30 nmol PE (*n* = 6), 30 nmol PE + 10 nmol idazoxan (*n* = 5), and 10 nmol idazoxan (*n* = 6).

In this section of the experiment, we further investigated the impact of idazoxan (α_2_-AR antagonist) on the antiallodynic effect of PE (30 nmol/rat) 14 days after pSNL. Intrathecal PE (30 nmol/rat) produced a significant antiallodynic effect, as indicated by significantly raised PWTs. Intrathecal co-administration of idazoxan (10 nmol/rat) inhibited the antiallodynic effect of PE (30 nmol/rat) 30 min after administration. On the other hand, the administration of idazoxan or the vehicle alone failed to affect PWTs of rats (Figure 3).

### 3.4. Oral Co-Administration of PE with PGB Acutely Alleviates Tactile Allodynia in Rats with Mononeuropathic Pain

**Figure 4:**
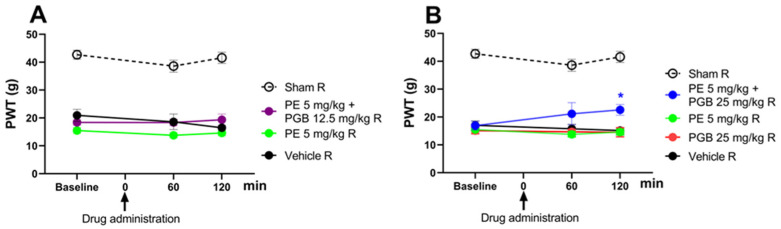
The antiallodynic effect of PE (5 mg/kg), PE/PGB (5 mg + 12.5 mg/kg), and vehicle (**A**); PE (5 mg/kg), PGB (25 mg/kg), PE/PGB (5 mg + 25 mg/kg), and vehicle (**B**) on pSNL-induced allodynia in rats after acute oral administration. The PWTs were measured on day 14 after pSNL at 60 and 120 min following oral administration. Data are given as mean ± SEM, *n* = 5–6 per group. The PWTs were compared before and after treatment (two-way ANOVA F (8, 69) = 1.097; Dunnett’s post hoc test, * *p* < 0.05 statistically significant compared to the vehicle-treated right (R) operated hind paw.

The effect of the combination of PE (5 mg/kg) and PGB (12.5 or 25 mg/kg) administered orally was investigated in rats that developed allodynia on day 14 after pSNL (Figure 4A,B). The combination of 5 mg PE and 12.5 mg PGB failed to produce an antiallodynic effect (Figure 4A). On the other hand, the 5 mg PE and 25 mg PGB combination significantly alleviated the tactile allodynia of rats with NP at 120 min (Figure 4B) after acute oral administration compared to the vehicle. In contrast, neither PE nor PGB alone had an effect, as shown at the measurement time points (Figure 4B). The 5 mg PE and 25 mg PGB combination is investigated further in Section 3.5.

### 3.5. Chronic Oral Co-Administration of PGB with PE Alleviates Tactile Allodynia in Rats with Mononeuropathic Pain

**Figure 5:**
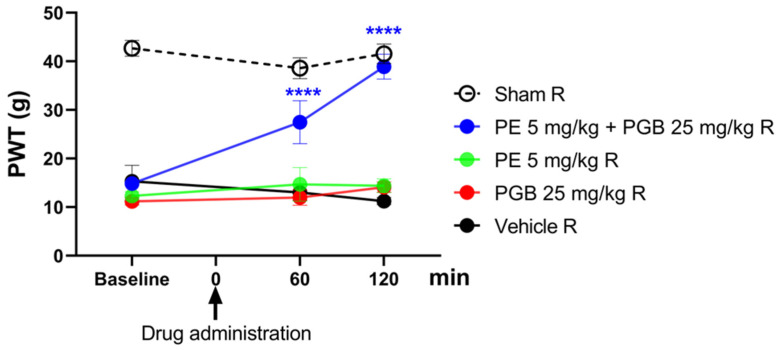
The oral antiallodynic effect of chronic PE or PGB alone, or their combination in rats with pSNL. The PWTs were measured on day 21 after 1 week of chronic treatment (twice daily), and DPA measured tactile allodynia at 60 and 120 min after oral administration. Data are expressed as mean ± SEM, *n* = 5–6 per group. The PWTs were compared before and after treatment (two-way ANOVA F (8, 69) = 6.667; Dunnett’s post hoc test, **** *p* < 0.0001 statistically significant compared to the vehicle-treated right (R) operated hind paw.

Figure 5 depicts the effect of oral PE (5 mg/kg) and PGB (25 mg/kg) administered alone or in combination (5 mg/kg and 25 mg/kg, respectively) on rats with pSNL-induced tactile allodynia, with the treatments given for one week twice daily. At baseline, 60 and 120 min, tactile allodynia is indicated by low PWT measured by DPA. The PE/PGB combination showed a significant antiallodynic effect at both time points, as indicated by an increase in PWT, while PE and PGB alone failed to produce an effect.

### 3.6. Effects of PE on [^3^H]NA Release from the Mouse Spinal Cord Ex Vivo Slice Preparation

**Figure 6:**
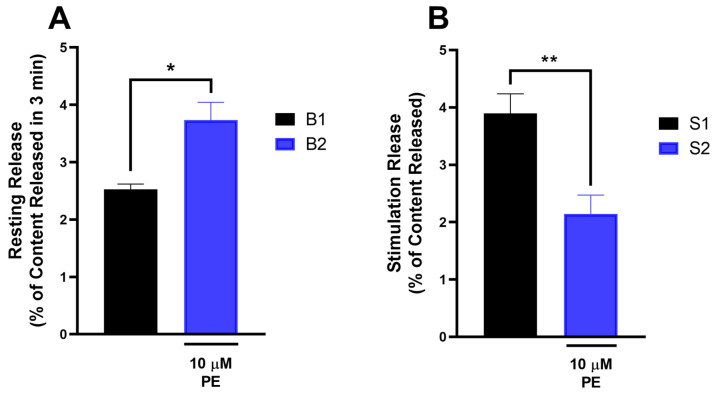
The effect of PE on the resting (**A**) and electrical stimulation-evoked (**B**) [^3^H]NA release in the mouse spinal cord. Data are expressed as mean ± SEM, *n* = 6 per group. (**A**) In case of resting release, fractional release was compared with and without PE (two-tailed *t*-test, *t* = 3.105, df = 5, with a 95% confidence interval from 0.2080 to 2.208). The effect size, as measured by partial eta squared, was 0.6585, indicating a large effect. * *p* < 0.05 statistically significant. The fractional stimulated release (**B**) was compared with and without PE (Paired two-tailed *t*-test, *t* = 5.564, df = 5, with a 95% confidence interval from −2.56 to −0.94). The effect size, as measured by partial eta squared, was 0.861, indicating a large effect. ** *p* < 0.01 statistically significant.

Figure 6 depicts that the presence of PE produced a significant increase in the release of [^3^H]NA from mouse spinal cord tissues under resting conditions (Figure 6A). On the other hand, PE inhibited the electrical stimulation-evoked release of [^3^H]NA from mouse spinal cord tissues (Figure 6B).

#### 3.6.1. Prazosin and Idazoxan Inhibit PE-Induced Smooth Muscle Contraction in the Mouse Vas Deferens Assay

**Figure 7:**
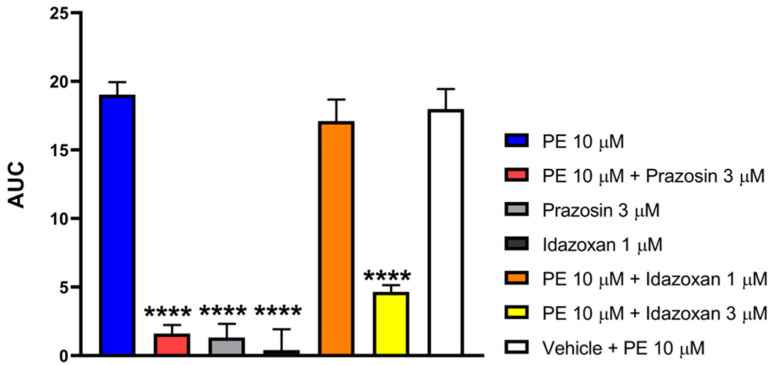
The effect of PE on MVD alone or in the presence of prazosin (3µM), idazoxan (1 and 3 µM), or the vehicle. The AUC values were calculated as the integral of the contraction curve relative to the baseline of 2 min for each contraction. Data are expressed as mean ± SEM, *n* = 7 per group. One-way ANOVA: F (6, 61) = 47.58, *p* < 0.0001, Dunnett’s post hoc test PE 10 μM + prazosin three μM *p* < 0.0001, PE 10 μM + idazoxan 3 μM, *p* < 0.0001, PE 10 μM + idazoxan 1 μM, *p* = 0.8113. (**** *p* < 0.0001 vs. PE 10 μM).

Figure 7 shows that PE in a concentration of 10 μM induced significant contraction in the MVD. The presence of 3 μM of either prazosin or idazoxan, but not 1 μM idazoxan or vehicle, antagonized the PE-induced contractions. Prazosin, idazoxan or the vehicle per se had no impact on MVD contractions. Statistical analysis was done using one-way ANOVA with Dunnett’s post hoc test.

#### 3.6.2. Idazoxan Inhibits the Effect of Clonidine on Electrical Field Stimulation-Induced Contraction in the Mouse Vas Deferens Assay

**Figure 8:**
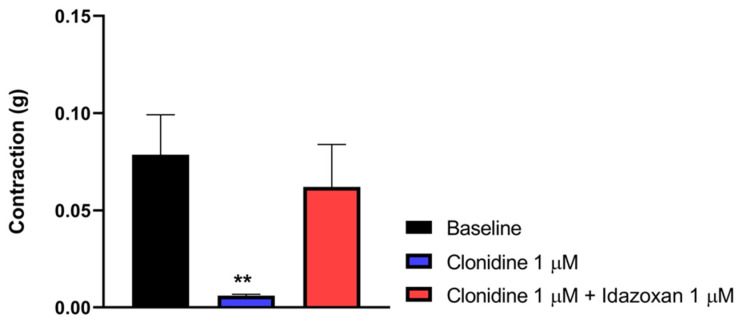
The effect of clonidine alone or in the presence of idazoxan (1µM) on MVD contraction. The contractions are given in grams. Data are expressed as mean ± SEM, *n* = 7 per group. One-way ANOVA: F (2, 18) = 4.821, *p* = 0.0211, Dunnett’s post hoc test, clonidine 1 µM *p* = 0.0156, idazoxan 1 μM, *p* = 0.72810. (** *p* < 0.01 vs baseline).

In this part of the experiment, we investigated the impact of idazoxan on the effect of clonidine, a selective α_2_-AR agonist, in the MVD assay. Figure 8 depicts that clonidine in a concentration of 1 µM abolished the muscle contraction of the MVD. This effect was reversed by 1 µM idazoxan. These data indicate that α_2_-ARs mediate the action of clonidine. One-way ANOVA analyzed the significance level between the groups with Dunnett’s post hoc test.

### 3.7. The Impact of Oral PE, PGB, and PE/PGB Combination on Motor Function in the Rat Rotarod Test

**Figure 9:**
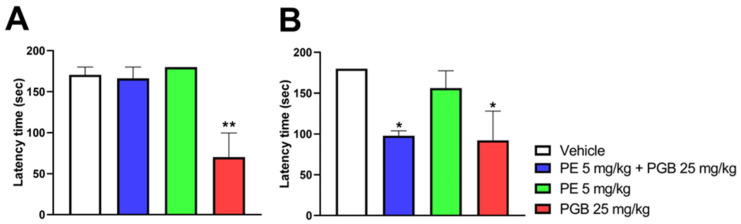
The acute motor coordination effects of PE (5 mg/kg) or PGB (25 mg/kg) per se, and PE/PGB combination on naïve rats 60 (**A**) and 120 (**B**) min after oral treatment. Columns represent the latency time of animals on the rotarod test. Data are expressed as mean ± SEM, *n* = 5 per group; analyzed by Kruskal–Wallis test with uncorrected Dunn’s post hoc test, 60 min: H = 10.11; 120 min: H = 8.060, * *p* < 0.05, ** *p* < 0.01 com-pared to the vehicle.

Figure 9 shows the impact of acute oral treatment with 5 mg/kg PE or 25 mg/kg PGB alone, or the combination of 5 mg/kg PE and 25 mg/kg PGB on rat motor function and coordination. PE alone did not affect rat motor function at either 60 min (Figure 9A) or 120 min (Figure 9B) after oral administration. In contrast to 5 mg/kg PE, 25 mg/kg PGB caused motor dysfunction at both time points. Finally, the combination of 5 mg/kg PE and 25 mg/kg PGB significantly affected the rat motor function, as indicated by the latency time on the rotating rod 120 min after oral administration (Figure 9A,B).

### 3.8. The Impact of PE, PGB and PE/PGB Combination on Arterial Blood Pressure

**Figure 10:**
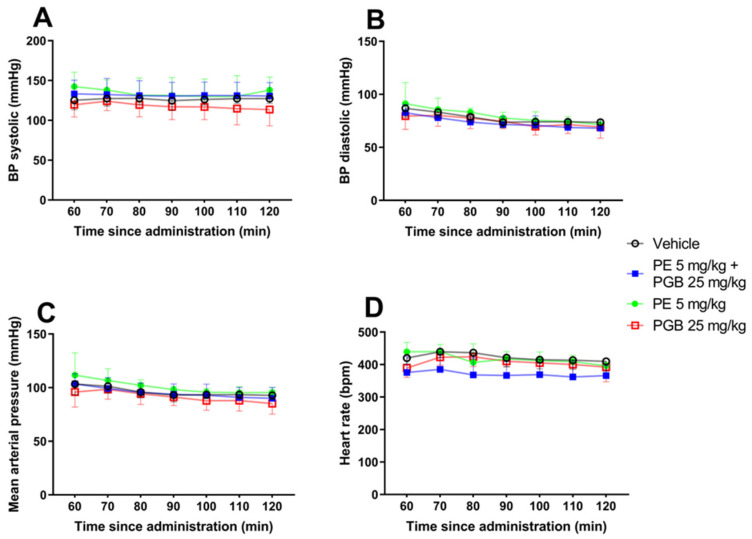
The effect of acute oral administration of 5 mg/kg PE, 25 mg/kg PGB, and PE/PGB (5 mg/kg + 25 mg/kg) on blood pressure and heart rate in naïve rats at different time points extending from 60 to 120 min: (**A**) systolic blood pressure F (18, 91) = 0.4322, (**B**) diastolic blood pressure F (18, 91) = 0.3241, (**C**) mean arterial pressure F (18, 91) = 0.3333 and (**D**) heart rate F (18, 90) = 1.327; two-way ANOVA, *n* = 4–5 per group.

Blood pressure analysis after acute oral administration of 5 mg/kg PE, 25 mg/kg PGB, or 5 mg/kg PE + 25 mg/kg PGB was carried out. Animals were anesthetized with isoflurane, and the right carotid artery was cannulated to measure blood pressure. No significant difference was observed between the treatment groups at different time points from 60 to 120 min: (A) systolic blood pressure, (B) diastolic blood pressure, (C) mean arterial pressure and (D) heart rate (Figure 10).

## 4. Discussion

Despite the availability of multiple mono- and combination pharmacotherapies for NP, achieving effective, rapid onset, and durable pain relief remains challenging.

Our findings demonstrated that PE produces an antiallodynic effect, whether tested alone or in combination with PGB, strongly reinforcing the notion that repurposing drugs holds significant potential for developing novel treatment approaches to manage NP. Intrathecal PE alone produces a pronounced antiallodynic effect. This allowed us to dissect the receptor mechanisms underlying PE-induced antiallodynic effect. In this context, we showed that the antiallodynic effect of intrathecal PE is abolished by both prazosin and idazoxan, which are considered selective antagonists of α_1_- and α_2_-ARs, respectively, indicating the involvement of α_1_- and α_2_-ARs. In fact, earlier in vivo studies from our group and other research groups have pointed to the effect of prazosin on α_2_-ARs [67,68,69]. The involvement of α-ARs aligns with our recent findings regarding the indirect action of PE, specifically its NA-releasing property. However, this finding also necessitates measuring the effect of PE on NA release from spinal tissues to support our hypothesis. One of the novelties of our current study is that PE has NA-releasing properties in neuronal spinal tissue, which may contribute to its antiallodynic effect.

A previous study by Nakai and co-authors has shown that intrathecal PE can alleviate the trigeminal pain developed after injury to the rat infraorbital nerve; however, in this study, the applied dose of PE was ten times larger than the doses applied in the current study [70]. The authors have attributed the antiallodynic effect of PE to its mediatory action on α_1_- and α_2_-ARs but did not address the potential indirect effects of PE. A recent study by Ohashi and coworkers has shown that both α_1_- and α_2_-ARs are involved in pain modulation [71]. In fact, the literature data also point to the presence of α_2_-AR subtypes, specifically α_2A-_AR in the dorsal horn of the spinal cord, which play a crucial relay point in the pain pathway [72,73,74,75]. Given this context, our study differs from previous studies in two aspects: first, the pain model utilized was a mononeuropathic pain model; second, the concept that PE can induce spinal cord neuronal NA release is an original one developed by our group. To the best of our knowledge, no data have been reported on the indirect spinal antiallodynic effect of PE thus far.

To decipher the mechanism, our ex vivo experiments provide direct neurochemical evidence that PE acts as an indirect NA releaser in the spinal cord. In these experiments, PE significantly enhanced resting [^3^H]NA release while simultaneously reducing electrically evoked NA release, consistent with depletion of releasable vesicular pools due to increased cytosolic NA efflux. This mechanism aligns with our previous findings demonstrating that PE functions as a substrate for the NET, inducing non-vesicular NA release [29]. These results suggest that the intrathecally injected PE releases NA, which can subsequently activate spinal ARs and modulate nociceptive transmission. Given the established role of NA in spinal pain inhibition, this mechanism warrants further investigation [76].

We have also extended our work to analyze the impact of PE on isolated MVD, which hosts different AR subtypes, thus highlighting the AR’s contribution to PE action [77]. The results obtained in MVD indicate that the effect of PE is mediated by both α_1_- and α_2_-ARs. However, in the same organ, our recent study has shown an impressive effect of PE, which is related to the NET-mediated mechanism [29]. Notably, PE can release NA from the cytosol of neuronal cells of MVD [29]. Additionally, an earlier in vitro study by Vizi and co-workers demonstrated that PE has an indirect effect, resulting from NA release, and highlighted the indirect effect of PE [78]. A study by Fabrizio Ledda & Laura Mantelli has shown that the effect of PE on guinea-pig isolated atria is partially reversed by either yohimbine or prazosin, which are considered selective antagonists of α_2_- and α_1_-ARs, respectively [79]. In clinical settings, prazosin has been shown to block the hypotensive effects of clonidine, a selective α_2_-AR agonist, in patients with essential hypertension [68]. Although prazosin is classically regarded as a selective α_1_-AR antagonist, several studies have demonstrated its ability to interfere with α_2_-AR-mediated responses under certain conditions [67,68,69]. In the context of pain, intrathecal prazosin has been shown to counteract the antiallodynic effects of selective α_2_-AR agonists [80], supporting the notion that prazosin-sensitive mechanisms may extend beyond α_1_-AR blockade.

Several studies supported that α_2_-ARs are densely expressed in the dorsal horn of the spinal cord, a critical site for nociceptive integration, whereas α_1_-ARs are more sparsely distributed in superficial laminae and enriched in deeper layers [81,82,83]. In addition, substantial evidence supports a central role of α_2_-ARs in spinal antinociception. Activation of presynaptic α_2_-ARs inhibits the release of excitatory neurotransmitters, including glutamate, from primary afferent terminals, thereby suppressing nociceptive signalling [84,85,86]. In animal models of NP, α_2_-AR agonists such as dexmedetomidine and ST-91 exert potent spinal antiallodynic effects [75,80,87,88,89]. Importantly, our data are likely to support α_2_-AR agonist-mediated antiallodynic action of PE. This claim relies on the fact that a previous study has shown that methoxamine, a direct α_1_-AR agonist, fails to exert an antiallodynic effect in rats with nerve injury-evoked NP, even at higher doses [80]. We should also acknowledge the previous works, where they showed that spinal NA can produce an antinociceptive effect through the activation of α_2_-ARs located on primary and secondary sensory afferent neurons, facilitating presynaptic and postsynaptic inhibition, respectively, as well as through the activation of α_1_-ARs located on inhibitory interneurons [90]. It means that, once NA activates α_1_-ARs located in the inhibitory interneurons, it halts pain. In this context, a study conducted by Seibt and Schlichter has convincingly shown that NA plays a significant role in modulating synaptic transmission within the deep laminae III-V of the spinal dorsal horn. Their findings indicate that both exogenously administered and endogenously released NA substantially enhance inhibitory synaptic transmission, specifically, the γ-aminobutyric acid (GABA)ergic and glycinergic pathways in laminae III-IV of the spinal dorsal horn [91]. This enhancement is mediated through the activation of α_1_-, α_2_-, and β-ARs. Since in our study the antiallodynic effect of PE was sensitive to α_1_- and α_2_-AR antagonists, it is presumably an α-AR-mediated effect. If we consider the above-mentioned results in relation to NP alongside data from Pertovaara, we anticipate that the released NA induced by PE would activate spinal α_1_, α_2_-ARs and consequently mitigate allodynia evoked by pSNL in rats.

Recent studies support the contribution of both α_1A_- and α_2_-ARs, which are involved in pain modulation [71,92]. Importantly, in the present study, in higher doses, PE showed acoustic startle reflex behaviours, corroborating a previous study on the effect of PE on this reflex after intrathecal administration [93]. It remains unclear whether the acoustic startle reflex behaviour is triggered by the activation of α_1_-AR, which are densely located in the deeper layers of the spinal cord; nevertheless, there is currently no data available concerning the subtypes of α_1_-AR [81,82]. In fact, the contradictory consensus on the role of α_1_-ARs in mediating antiallodynic effects at the spinal level underscores the need for future investigations to elucidate the contribution of spinal α_1_- and α_2_-ARs to the antiallodynic effects of PE, despite the data mentioned above and the observed failure of methoxamine, an α_1_-AR selective agonist, to produce antiallodynic effects.

In chronic pain conditions, specifically in NP, an imbalance exists between the excitatory and inhibitory systems [41,48,94,95,96]. In the present scenario, NA-evoked by PE could strengthen the descending pain pathway and eventually lower NP by activating spinal α_2_-ARs, similar to the effects observed with current drugs that are used to manage NP, such as TCAs, SNRIs, and others [7,35,94].

After establishing the possible PE target in the spinal cord in the context of the implicated mechanism, we moved on to the next step, to investigate the impact of oral PE in combination with PGB on NP after acute and long-term treatments. The question may arise as to why our choice is based on these drugs and doses that make up the combination. PGB produces an antiallodynic effect by modulating spinal α_2_δ1-containing voltage-gated calcium channels. In fact, as mentioned in the introduction, PGB is considered a first-line medication for the treatment of NP, but a high NNT indicates that NP management has not yet been resolved [5]. Generally, combination therapy in the management of NP is aimed at increasing therapeutic efficacy and reducing side effects or both [17]. Thus, we presumed that combining PGB with a novel or repurposed drug such as PE can enhance its antiallodynic effect, while potentially decreasing side effects.

In this series of experiments, based on the toxicology and carcinogenesis studies of PE hydrochloride issued by the U.S. Department of Health and Human Services, we chose the oral dose of 5 mg/kg to investigate its antiallodynic effect either alone or in combination with PGB [97]. The 25 mg/kg dose of PGB was chosen based on our previous work and others [48,97]. The selected PGB dose did not show an anti-tactile allodynic effect in rats with mononeuropathic pain either after acute or chronic treatment in accordance with our previous work [48]. Likewise, PE alone did not exert anti-tactile allodynic effects after either acute or chronic treatments. Concerning PE, to the best of our knowledge, there is no data on its oral effect against mononeuropathic pain. However, data on its oral low bioavailability (38%) exists [98]. On the other hand, PGB exhibits predictable pharmacokinetics, with an absolute bioavailability of approximately 90%, as measured by the recovery of unchanged drug in urine [99]. Significantly, the combination of PE and PGB exerted antiallodynic effects after both acute and chronic treatments, and with a rapid onset. Our results suggest that the combination of drugs strengthens the spinal adrenergic system, and drugs modulating spinal α_2_δ1-containing voltage-gated calcium channels may offer therapeutic benefits. The concept of combination therapies utilizing medications with different pharmacodynamic actions aims to address the limitations of analgesic monotherapy by providing augmented analgesic effects at lower individual drug doses. A preclinical study has evaluated the impact of PGB combined with duloxetine, venlafaxine, and tramadol in rats with NP [100]. In this study, the combination of PGB with duloxetine or tramadol produced an antiallodynic effect greater than that of the individual drugs administered alone. On the other hand, the PGB–venlafaxine combination produced a diminished effect compared to that of the individual drugs, indicating a potential antagonistic pharmacodynamic interaction. These data collectively underscore the importance of investigating drug–drug interactions in preclinical models prior to clinical translation. The mechanistic similarity between agents such as duloxetine and venlafaxine, in terms of their capacity to improve the impaired descending noradrenergic inhibitory system, does not necessarily predict equivalent interaction profiles when either agent is combined with a third pharmacological agent, such as PGB. Triple combination therapy with PGB, duloxetine, and tramadol was investigated in mice with NP, and it was found to produce superior antiallodynic effects compared with the individual monotherapies or dual combinations, without evidence of motor impairment [101]. Clinical data on the combination of PGB and duloxetine at moderate doses demonstrated similar efficacy and a comparable side effect profile to each drug administered as monotherapy [5]. Indeed, evidence and expert consensus recommendations support the combination of gabapentinoids with SNRIs as a superior therapeutic strategy, yielding greater analgesic efficacy and a more favourable side effect profile compared with high-dose monotherapy alone [5]. The COMBO-DN clinical study, in which either PGB or duloxetine alone, or their combination, was administered to patients who had not responded to standard doses of duloxetine or PGB, demonstrated the non-superiority of this combination over high-dose monotherapy for the treatment of NP in diabetes, with no significant differences in treatment-emergent adverse events observed among the treated groups [53].

Thus, there is still great clinical relevance for the treatment of poorly PGB-responsive NP, in whom dose escalation of PGB is limited by increased adverse side effects. The significance of the present study lies in the finding that NP was significantly inhibited by the combination of PGB and PE, with the greatest antiallodynic effect of rapid onset that was not observed in response to treatment with the individual drugs alone. These data are promising in terms of the antiallodynic effect of the combination; however, future studies are required to further elucidate the advantages and disadvantages of this combination before proceeding to the clinical setting.

Several studies have reported on the side effects of PGB in the context of motor function, cardiovascular function, sleepiness, and other conditions. Evidence indicates that higher doses of PGB can cause impairments in coordination and ataxia, although this effect varies based on dosage and context [48]. Nevertheless, contradictory results have been reported, and some of these studies have linked the side effects to high doses of PGB [48,102,103]. Both PGB and the PE/PGB combination, administered at doses of 25 mg/kg and 5/25 mg/kg, respectively, altered rat motor performance. Indeed, the oral dose of 25 mg/kg of PGB was found to have no significant antiallodynic effect but exhibited side effects similar to those of the 5/25 mg/kg PE/PGB combination. Our previous study on the antiallodynic effect of PGB in rats demonstrated that PGB at a dose of 50 mg/kg produced an antiallodynic effect only following chronic treatment—specifically, after two weeks of administration [48]. The tested dose of PGB was associated with impairment in motor function. In the present study, however, 25 mg/kg of PGB, when combined with PE, produced a stronger antiallodynic effect with a notably faster onset. Consequently, the applied dose of PGB was halved relative to that employed in the previous work, demonstrating that an antiallodynic effect can be achieved at a lower dose when PGB is combined with PE. Importantly, the impact of PGB in NP varies, depending on NP type, the route of administration, the doses applied and analgesic assay [104,105,106]. Our current findings have been confirmed regarding the effect of the PE/PGB combination in a preclinical scenario, but their effectiveness in real-world clinical settings remains uncertain and requires further evaluation. As mentioned above, a study on the effects of PE on motor disturbance found that intrathecal PE at higher dosages can cause acoustic startle; however, in our current study, no such behaviours were observed following oral administration of PE alone or the combination [93].

Next, the study aimed to evaluate the impact of the PE, PGB and their combination on the rat heart rate and blood pressure. In this regard, there is disagreement between preclinical and clinical research regarding how systemic PGB affects systolic blood pressure and pulse rate; it may or may not result in a notable decrease [107,108]. In contrast to motor function, the combination dose or the single drug doses indicate that we could potentially achieve an antiallodynic effect while minimizing cardiovascular side effects because neither the combination nor the single drugs altered blood pressure or heart rate under our current experimental setup. As a future task, measuring these parameters in non-anesthetized rats using noninvasive techniques would be more convincing in determining whether isoflurane impacted the measured parameters or not.

Finally, clinical studies are warranted to assess the safety, efficacy, and optimal dosing of the PE/PGB combination in clinical settings.

Limitations: The current study was focused on the pharmacodynamic profile of PE when applied as a single drug or in combination with PGB. Issues arise about the pharmacokinetics profile, since the absorption of PE after oral administration in humans is variable, and the FDA stated that PE is not effective following this route of drug administration. We can prove that PE can be absorbed after oral administration only through experiments, and based on the results, we can support or oppose the published data on PE bioavailability. We have no data on the impact of PGB on the absorption of PE. Future research is therefore required to address each of these concerns. As future perspectives, it is important to evaluate the oral PGB and parenteral PE combination, which may offer realistic bioavailability. This combination could be pivotal for initiating therapy, given that current first-line medications for NP often have a slow onset. Furthermore, determination of the quantity of spinal NA using the microdialysis technique, as well as the contribution of the α-AR subtypes to PE-induced antiallodynic effects, is crucial for fully understanding the mechanism of PE before proceeding to clinical testing.

## 5. Conclusions

We identified that injection of PE into the spinal cord produces a measurable antiallodynic effect of fast onset against mononeuropathic pain. PE-induced analgesia by releasing NA, which activates spinal α_1_- and α_2_-ARs. Targeting both α-ARs and α_2_δ-containing voltage-gated calcium channels significantly reduced pSNL-induced tactile allodynia acutely and chronically following systemic administration. Notably, the antiallodynic effect of PE alone or in combination with PGB is of fast onset, making it a novel strategy to treat NP. The combination of PE and PGB does not worsen motor function and is devoid of cardiovascular side effects after acute administration.

## Data Availability

The datasets produced and analyzed during this study are available upon request.

## Abbreviations

The following abbreviations are used in this manuscript:

NP	Neuropathic pain
TCAs	Tricyclic antidepressants
NNT	Number needed to treat
PE	Phenylephrine
AR	Adrenoceptor
MVD	Mouse vas deferens
NA	Noradrenaline
NET	Noradrenaline transporter
SNRIs	Serotonin–norepinephrine reuptake inhibitors
PGB	Pregabalin
pSNL	Partial sciatic nerve ligation
PWTs	Paw withdrawal thresholds
DPA	Dynamic plantar Aesthesiometer
